# Intrauterine Inflammation Leads to Select Sex- and Age-Specific Behavior and Molecular Differences in Mice

**DOI:** 10.3390/ijms24010032

**Published:** 2022-12-20

**Authors:** Ana G. Cristancho, Natalia Tulina, Amy G. Brown, Lauren Anton, Guillermo Barila, Michal A. Elovitz

**Affiliations:** 1 Division of Child Neurology, Department of Pediatrics, Children’s Hospital of Philadelphia, Philadelphia, PA 19104, USA; 2 Department of Neurology, Perelman School of Medicine at the University of Pennsylvania, Philadelphia, PA 19104, USA; 3 Department of Obstetrics and Gynecology, Perelman School of Medicine at the University of Pennsylvania, Philadelphia, PA 19104, USA

**Keywords:** intrauterine inflammation, behavior, sex, age, lipopolysaccharide, neurogenesis

## Abstract

Sex-specific differences in behavior have been observed in anxiety and learning in children exposed to prenatal inflammation; however, whether these behaviors manifest differently by age is unknown. This study assesses possible behavioral changes due to in utero inflammation as a function of age in neonatal, juvenile, and adult animals and presents potential molecular targets for observed differences. CD-1 timed pregnant dams were injected in utero with lipopolysaccharide (LPS, 50 μg/animal) or saline at embryonic day 15. No differences in stress responses were measured by neonatal ultrasonic vocalizations between LPS- and saline-exposed groups of either sex. By contrast, prenatal inflammation caused a male-specific increase in anxiety in mature but not juvenile animals. Juvenile LPS-exposed females had decreased movement in open field testing that was not present in adult animals. We additionally observed improved memory retrieval after in utero LPS in the juvenile animals of both sexes, which in males may be related to a perseverative phenotype. However, there was an impairment of long-term memory in only adult LPS-exposed females. Finally, gene expression analyses revealed that LPS induced sex-specific changes in genes involved in hippocampal neurogenesis. In conclusion, intrauterine inflammation has age- and sex-specific effects on anxiety and learning that may correlate to sex-specific disruption of gene expression associated with neurogenesis in the hippocampus.

## 1. Introduction

Clinical data strongly support the link between maternal infections during pregnancy and a wide range of adverse neurobehavioral outcomes in exposed children, such as learning delay, cerebral palsy, depression, anxiety, schizophrenia, epilepsy, attention-deficit disorder, and autism [1,2,3,4]. These neurological abnormalities induced by intrauterine inflammation are thought to result from cellular and molecular alterations that lead to grey and white matter injury in the developing brain [5,6,7,8]. Experimental animal models have suggested that activation of the innate immune response causes long-lasting changes in brain function and behavior [9,10]. In models of maternal systemic inflammation, previous studies have shown that the activation of the Toll-like receptor (TLR) signaling pathway during pregnancy leads to alterations in certain behavioral outcomes [6,7,8,9,10,11,12,13,14]. However, systemic maternal inflammation is only one mechanism by which prenatal inflammation can occur and it does not recapitulate the local inflammation that is frequently present in preterm birth [15,16].

Here, we use a mouse model of localized intrauterine inflammation to more accurately recapitulate a common clinical scenario by which a fetus is exposed to inflammation [16]. This model has been shown to produce molecular and cellular hallmarks of fetal and postnatal brain injury [16,17,18,19,20]. With this model, we can assess possible behavioral alterations resulting from prenatal infection and inflammation as modeled by in utero exposure to a sterile inflammatory stimulus (lipopolysaccharide, LPS). Using this model, we previously reported that exposure to intrauterine inflammation did not result in sex-specific differences in anxiety in 12–14-week-old mice [21]. However, recent evidence demonstrates that sex and age both independently and synergistically alter anxiety-related behaviors and learning. Significant differences are sometimes observed in juvenile mice that are absent in adult animals [22,23,24,25]. In addition, some sex-specific differences exist in early neurodevelopmental milestones. For example, negative geotaxis males are slower to turn around when placed on an incline in the first 10 days of life [26].

While these studies demonstrate sex-specific behavioral development patterns, no studies have investigated the interaction between the effects of intrauterine inflammation with sex throughout this age span on anxiety and learning. Here we compare the development of a stress-induced anxiety response and animals’ performance in spatial learning and memory tasks in the offspring of dams exposed to in utero inflammation or saline during pregnancy. In addition, since anxiety-related behaviors and learning and memory are known to be influenced by changes in hippocampal neurogenesis [27,28,29,30,31], we also determined if the expression levels of genes associated with adult neurogenesis were altered in response to LPS exposure in male and female pups. A better understanding of how prenatal inflammation affects behavior in exposed offspring in a sex-specific manner and over a lifetime is important for developing new therapeutic approaches to improve inflammation-induced adverse neurobehavioral outcomes.

## 2. Results

### 2.1. Intrauterine Inflammation Does Not Alter Ultrasonic Vocalizations in Neonatal Pups

**Figure 1:**
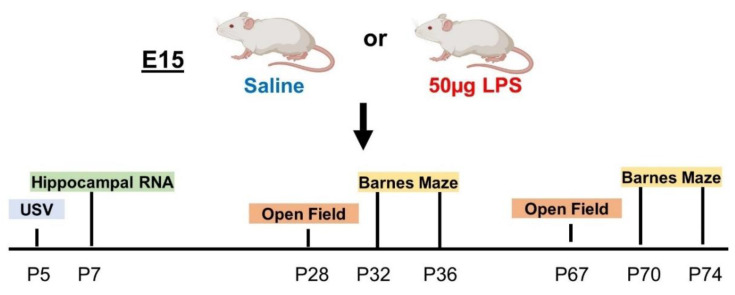
Schematic and timeline of experiments. Created with BioRender.com.

**Figure 2:**
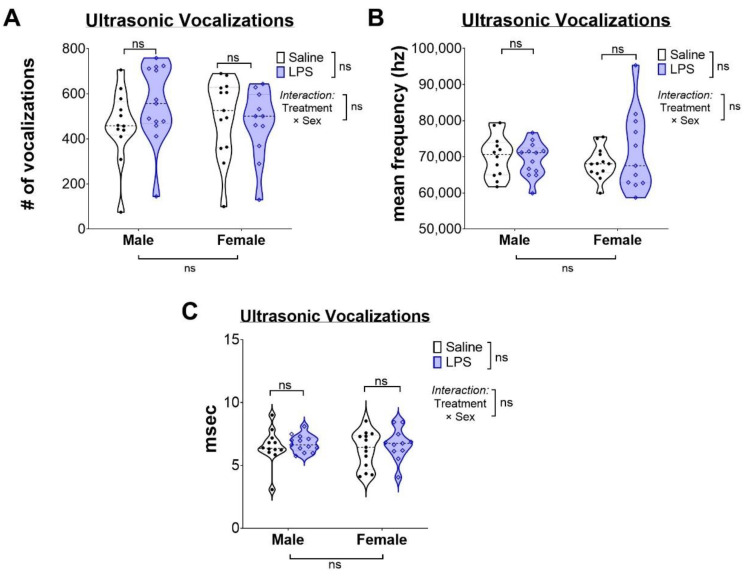
Intrauterine inflammation is not correlated to changes in USV. Violin plots quantifying the number (**A**), frequency (**B**), and duration (**C**) of USV. Individual points represent a single animal. “ns” indicates “not significant.”

After exposure to an intrauterine LPS insult, we performed a battery of behavior tests throughout the lifespan (Figure 1). USV testing in the neonatal period was performed to study the pups’ response to the stress of separation from their mothers and as an assessment of early deficits in communication [32]. Anxiety-related phenotypes are associated with increased vocalizations, whereas autism-related phenotypes are associated with decreased vocalizations [32]. Therefore, we performed USV testing on saline- and LPS-exposed male and female pups on P5 isolated from their mothers as an early test for neurodevelopmental deficits. Vocal responses resulting from the isolation from the mother, specifically, the total number of calls per five-minute interval (Figure 2A), the frequency of emitted sound (Figure 2B), and the duration of the calls (Figure 2C) were compared between the groups. Our data show no significant changes in vocal responses between saline and LPS groups in male or female animals. Thus, LPS exposure does not affect social communication skills, at least at this early stage of postnatal development.

### 2.2. Intrauterine Inflammation Leads to Increased Anxiety-Related Behavior in Adult, but Not Juvenile, Males

**Figure 3:**
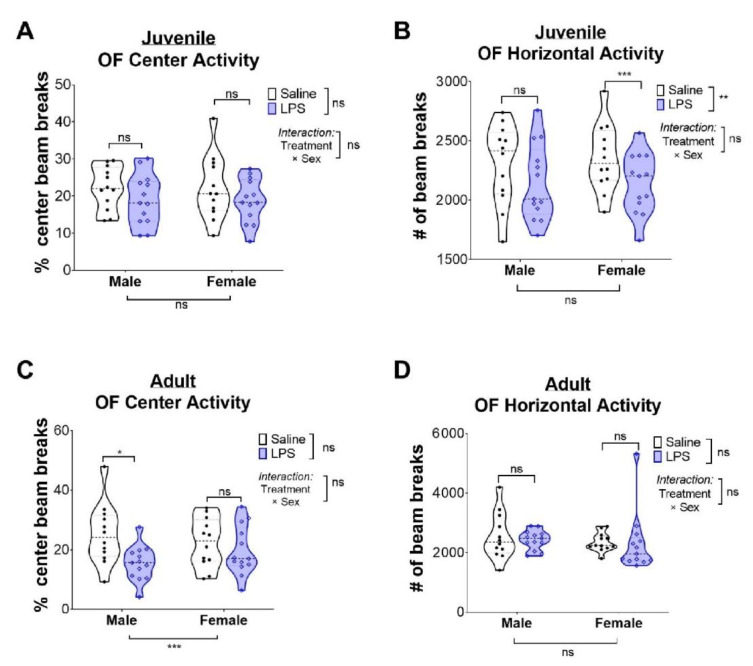
Adult males have increased anxiety-like behaviors after exposure to intrauterine inflammation. Violin plots quantifying the (**A**) percent of center beam breaks in open field and the (**B**) extent of total movement in juvenile animals in OF. In (**C**,**D**), these same parameters are quantified in adult animals. Individual points represent a single animal. Statistical significance was set at * *p* < 0.05, ** *p* < 0.01, and *** *p* < 0.001; “ns” indicates “not significant.”

We conducted OF testing to determine if intrauterine inflammation altered exploratory behaviors in a new environment in juvenile, pre-adolescent (P28), and mature (P67) animals. Decreased exploration of the center of an open field is associated with increased anxiety-related deficits [33,34,35]. LPS exposure did not increase time spent in the center of an open field for either sex in juvenile animals (Figure 3A). However, female juvenile mice exposed to LPS had decreased horizontal activity (Figure 3B), reflective of a possible subtle motor deficit. By contrast, LPS exposure significantly reduced the time male adult mice spent in the open field center, consistent with increased anxiety (Figure 3C). Neither sex had deficits in motor activity from LPS (Figure 3D), consistent with the resolution of the motor deficit observed in juvenile female mice from LPS.

### 2.3. Intrauterine Exposure to Inflammation Is Not Associated with Improved Memory in Juvenile Mice, but Impaired Memory in Adult Female Mice

**Figure 4:**
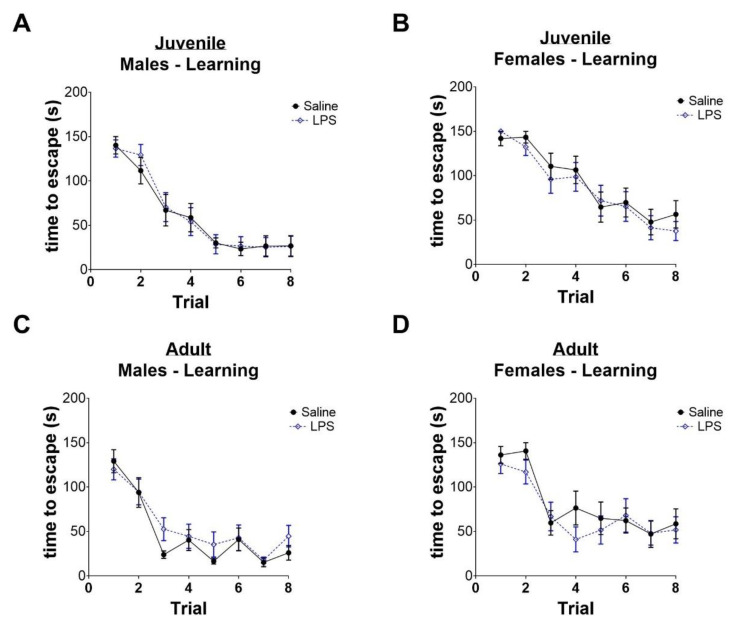
Intrauterine inflammation does not affect spatial learning in juvenile or adult mice. (**A**–**D**) Time to escape in juvenile and adult animals in each trial.

We next sought to test whether exposure to intrauterine inflammation predisposed animals to spatial learning and memory deficits. We used the BM test in juvenile (P32–36) and adult (P70–P74) animals. Male and female pups were tested as separate groups. Spatial learning is tested by using visual cues to direct the animals towards an “escape hole” in the apparatus. As expected, over the course of the acquisition trials, the male and female animals of both age groups were able to escape the apparatus more quickly, reflecting typical spatial learning (Figure 4A–D). There was no difference in the rate of learning between saline-exposed and LPS-exposed animals.

**Figure 5:**
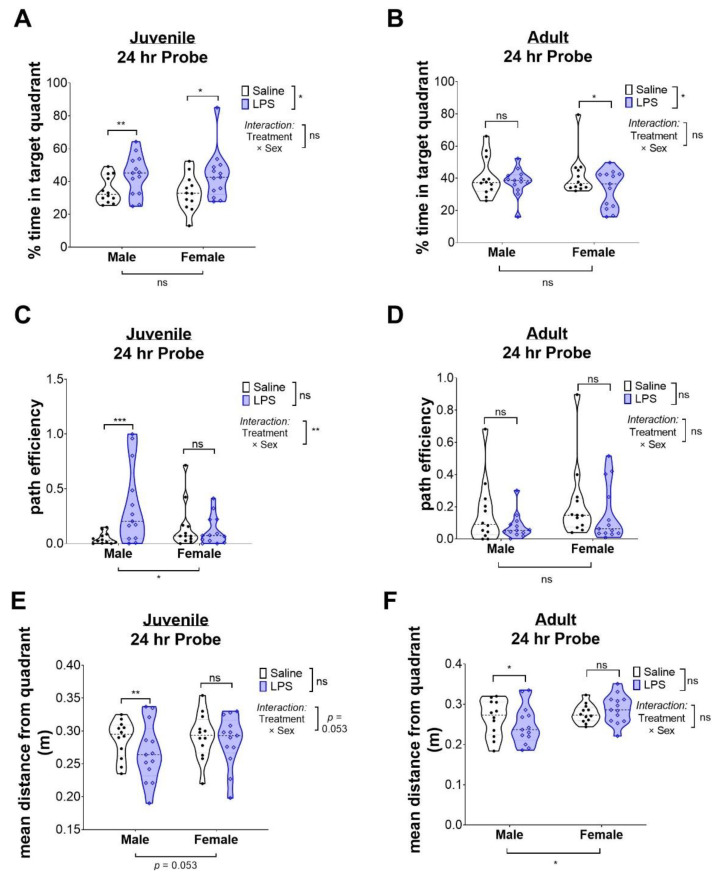
Intrauterine inflammation leads to disrupted memory patterns in juvenile and adult animals. (**A**,**B**) Time to escape in juvenile and adult animals. (**C**,**D**) Path efficiency to target quadrant. (**E**,**F**) Percent time spent in target quadrant for long-term memory recall studies in juvenile and adult mice. Individual points represent a single animal. Statistical significance was set at * *p* < 0.05, ** *p* < 0.01, and *** *p* < 0.001; “ns” indicates “not significant.”

However, substantial differences were found in the twenty-four-hour probe trial that may suggest differences in memory. Surprisingly, male and female juvenile mice, exposed to intrauterine inflammation, spent more time in the target quadrant than saline-exposed animals (Figure 5A), suggesting an improvement in memory. By contrast, in adult mice, there were no differences in male mice by exposure, but female mice exposed to intrauterine inflammation had a significant decrease in time in the target quadrant (Figure 5B), suggesting a sex-dichotomous reduction in long-term memory.

BM probe testing can also be used to assess more subtle differences in direct learning and cognitive flexibility [36]. To test if memory differences were associated with how directly the animal recalled the quadrant, we assessed the path efficiency of the animals during the probe trial. Path efficiency tests how rapidly and directly an animal reaches the target quadrant as another direct measurement of memory. Consistent with memory probe data, juvenile males exposed to LPS had increased path efficiency (Figure 5C). Notably, females did not have the same increased efficiency despite having a similar, albeit more variable, increase in memory after LPS exposure (Figure 5D). By contrast, path efficiency did not appear to be affected in adult animals by treatment, although there was significant variability in these samples.

We also tested if differences in long-term memory were associated with differences in how the animals explored the maze. The mean distance from the quadrant is how likely the animals are to stay in the same quadrant when unable to find the target; ideally, when unable to rapidly locate the target, the animal will eventually start to explore for a new escape area farther from the target. Staying closer to the target suggests some issues in cognitive flexibility. Notably, juvenile and, to a lesser extent, adult males treated with LPS were less likely to explore for a new target, suggesting LPS may lead to a perseverative phenotype that needs to be further explored (Figure 5E,F). Together, these data demonstrate there are sex-specific differences in memory and exploration, but also highlight the substantial variability seen across animals for all of these parameters.

### 2.4. Intrauterine Exposure to Inflammation Leads to Sex-Dichotomous Differences in Gene Expression Related to Neurogenesis

**Figure 6:**
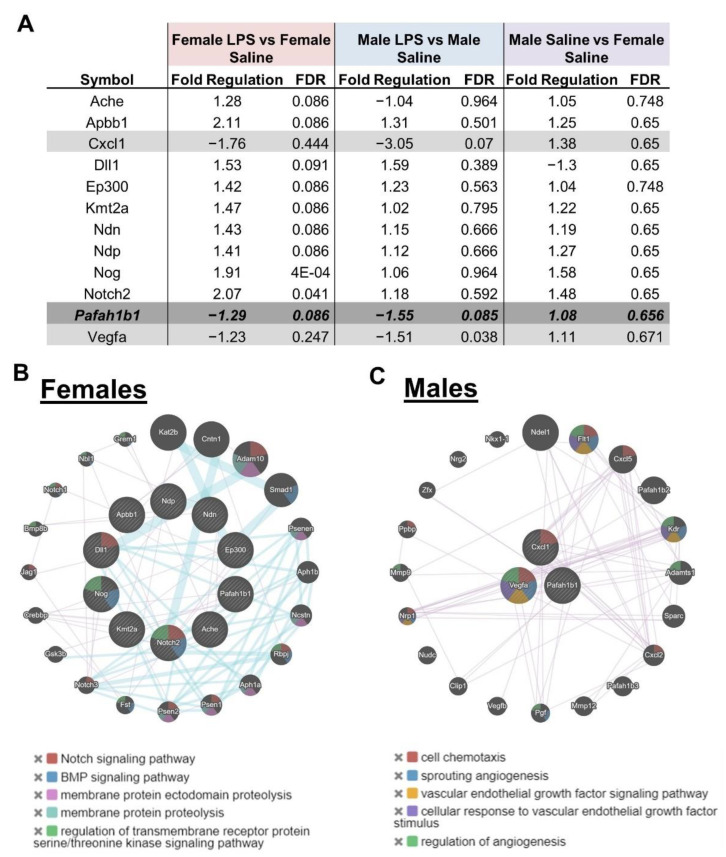
Sex dichotomies in neonatal hippocampal gene expression from intrauterine inflammation. (**A**) Table of neurogenesis-related genes that are dysregulated in the P7 hippocampus. Genes in white change in females only. Genes in light grey change in males only. Pafah1b1, in dark grey, is the only gene that changed in both males and females. GeneMania pathway analysis in genes differentially expressed in (**B**) females and males (**C**) (genes in the inner circle). Colored shading corresponds to the top five pathways enriched by an FDR <5%.

We had previously shown that at P7 there is already decreased hippocampal neurogenesis after intrauterine inflammation [37], so we sought to determine if there were sex-related differences in gene expression related to neurogenesis that may account for later differences in learning at this early time point. Expression levels of 84 genes known to play important roles in mouse neurogenesis were compared in the hippocampi of saline- and LPS-exposed pups using commercial RT-PCR arrays on P7. This analysis revealed there were hippocampal transcripts altered in response to prenatal inflammation in females only, in males only, or in both sexes when using an FDR <0.10 as a cut off (Figure 6A). Most of these changes were less than two-fold, except for Amyloid β A4 precursor protein-binding (Apbb1) and Notch gene homolog 2 (Notch2) genes in intrauterine-inflammation-exposed females. There were nine genes that only changed in females (white), two genes that only changed in males (light grey), and one gene, *Pafah1b1,* that changed in both sexes with LPS (dark grey). Of note, there were no differences in hippocampal neurogenesis gene expression between males and females in saline-exposed mice.

Previous research has demonstrated that intrauterine inflammation regulated neurogenesis [37]. To determine if there were sex-dichotomous neurogenesis-related pathways modulated by intrauterine inflammation, we used GeneMania to build weighted interaction networks based on differentially expressed genes. Genes regulated by intrauterine inflammation in female mice were enriched for the Notch and BMP pathway (Figure 6B). By contrast, the few genes regulated in males were related to chemotaxis and angiogenesis (Figure 6C). These findings suggest there may be sex-dichotomous pathways related by intrauterine inflammation in the hippocampus that may be accounting for sex differences in behavior.

## 3. Discussion

Exposure to intrauterine inflammation is a risk factor for both preterm birth and a spectrum of neurological, cognitive, and sensory-motor deficits manifested in exposed children during childhood and later in life [1,2,3,4]. Although there are ongoing efforts to establish the extent intrauterine inflammation leads to persistent disturbances in children [38], even in animal studies, relatively little is known about how the age and sex of the offspring of exposed mothers affect these adverse neurobehavioral outcomes. In this study, we utilized an established mouse model of intrauterine inflammation to show that in utero exposure to sterile inflammation can cause distinct behavioral changes in exposed offspring that are sex specific but also manifest differently throughout their lifetime. In particular, our data demonstrate that mature but not juvenile male pups exposed to in utero inflammation have elevated anxiety levels when transitioned to the OF. In addition, we observed a temporary improvement in the retrieval of spatial memory in juvenile pups of both sexes after exposure to inflammation; however, this behavioral change was not detected in older LPS-exposed animals suggesting age-specific evolution of memory. Indeed, female adult animals exposed to LPS had worse long-term memory. Lastly, prenatal inflammation altered expression levels of multiple genes involved in hippocampal neurogenesis. Interestingly, some altered transcripts showed sex-specific changes, while others were dysregulated in both male and female samples. These changes in expression levels are present during the early postnatal period (P7) and, therefore, may underlie, at least in part, the observed behavioral alterations. While we recognize there are limitations in interpreting animal behavior studies as a proxy for neurodevelopmental outcomes seen in humans because of the complexity of human outcomes, appropriately powered and without excessive reductionism of the results, animal studies still serve as an important basis of mechanistic studies when human studies are limited [39]. We have elected to display all the data points to facilitate interpretation and promote rigor and reproducibility by demonstrating the inherent variability of these studies.

Epidemiological studies have shown that inflammation during pregnancy is associated with a higher incidence of autism spectrum disorders in exposed children [7]. The link between prenatal inflammation exposure and the symptoms of autism was further demonstrated by using experimental animal models in which an innate immune response was induced prenatally by injecting bacterial or viral ligands systemically [40,41,42,43,44]. In this study, we addressed a possible effect of localized intrauterine inflammation on the development of social communication skills during the early postnatal period by using the USV test. In contrast to previous reports showing altered USV responses after systemic exposure to inflammatory stimuli [41,42,43,44], we did not reveal any differences between LPS- and saline-exposed groups in either sex. However, it should be noted that there is some disagreement regarding the effect of inflammation on USV responses; studies have shown both increases and decreases in vocalization after exposure to an inflammatory agent [40,41,42,43,44]. These discrepancies could result from using different mouse strains or various ages since both factors affect the behavioral performance in the USV test. For example, in the BTBR strain, exposure to prenatal inflammation increased the number of calls at P8, P10, and P12. By contrast, similar changes in vocalization response in C57/B6 mice exposed to the same inflammatory stimulus were detected only at P10 [45]. Our study examined social behavior in another mouse strain (CD-1) at an earlier time point (P5) and showed no difference between LPS- and saline-treated groups. This, however, does not exclude the possibility of behavioral alterations in these animals later during postnatal development.

Increased anxiety is associated with many neurological conditions linked to prenatal inflammation, including depression, autism, epilepsy, and schizophrenia [46,47]. We addressed whether the effect of prenatal inflammation on stress-induced exploratory behavior and anxiety levels depends on the sex of exposed offspring when examined at two different time points throughout their lifetime. Our data, obtained using the OF test, revealed that exposure to prenatal inflammation caused increased anxiety in LPS-exposed mature (P67) but not pre-adolescent (P27) male pups. However, female pups from both age groups remained unaffected. An increase in anxiety levels in LPS-exposed male pups detected in our study is consistent with previously published data which were obtained using both systemic immune activation [48,49,50,51,52,53,54,55,56], intrauterine exposure to infectious agents or their ligands [21,57], and prenatal hypoxia [58]. For instance, using the OF test, Dada, and co-authors showed that mouse offspring that received intrauterine LPS injections at E17 had elevated anxiety levels at P60, although sex-specific effects were not tested [57]. Other work has shown that in utero inflammation leads to higher anxiety in male and female pups between P84 and P98 [21]. Therefore, it appears sex and age play important roles in altering stress-induced anxiety in pups exposed to prenatal inflammation, though there is significant variability in the results.

We also determined if spatial learning and memory, behaviors that can be readily tested in mice, were altered in LPS-exposed pups. Our data showed that a mouse’s ability to learn and acquire spatial memories after exposure to LPS did not change compared with a saline control by sex or over the lifespan. In contrast, memory retrieval was moderately improved in LPS-exposed pups of both sexes at a younger age (P27), but in males, in particular, seemed to be due to a lifelong increase in preservative behaviors. The fluctuating nuances of the phenotype were clearer with multiple time points, which speaks to the importance of timing at different points of development to characterize the impact of the insult fully. We had some challenges finely analyzing the movements between different target areas in the BM because of tracking malfunctions of white mice on a white background but hope to repeat this experiment with higher contrasting apparatus. Furthermore, mechanisms underlying these fluctuating changes and possible perseverative behaviors warrant future studies.

Spatial learning and memory functions localize primarily to the hippocampus [59,60,61]. The execution of these behavioral tasks relies on hippocampal granule neurons produced both before birth and throughout adulthood in the hippocampal subgranular zone (SGZ) [62]. Interestingly, silencing of adult-born granule neurons using an optogenetic approach impairs memory retrieval but not memory acquisition [63], indicating that novel neuronal production is exclusively responsible for memory retention. Furthermore, adult neurogenesis can be modulated by various environmental and physiological stimuli, including the inflammatory status of the organism [64,65]. Our group demonstrated that intrauterine inflammation leads to a reduction in the rate of hippocampal neurogenesis between P7 and P14 and a subsequent decrease in total neuronal density at P28 [37]. Remarkably, these alterations in neuronal production in the hippocampal SGZ appear to take place at around the same time in postnatal development when an improvement in memory retrieval was observed in this study (P27). Previous data show that severe reduction and complete ablation of the hippocampal neurogenesis, caused by antimitotic agents and irradiation, respectively, can result in an impaired ability to form and retrieve spatial memories [66,67]. In contrast, our mouse model of intrauterine inflammation shows a modest decrease in the neurogenesis rate [37], which is associated with age- and sex-specific memory improvement in young males. This suggests a more nuanced relationship between the nature of an environmental insult, the degree of the consequential change in neurogenesis, and the behavioral response in affected offspring.

The mechanism by which the decrease in hippocampal neurogenesis could facilitate memory retrieval may involve an increase in the electrical activity of the remaining granule cells as compensation for the function of missing neurons. There is evidence that hippocampal granule neurons from pups exposed to in utero inflammation have increased excitatory synaptic strength [68], suggesting that these neurons are excited more frequently than those from inflammation-free pups. In addition, other brain areas and molecular pathways may also be responsible for the modulation of memory retrieval after exposure to prenatal inflammation. For instance, it has been shown that infants who survived clinical infections before birth show aberrantly increased cortisol levels [69,70]. While stress-induced cortisol response is known to both enhance and reduce memory performance [71], data show that a low level of cortisol found in the aging population correlates with a decline in spatial memory [72]. Therefore, an inflammation-induced increase in cortisol level may have an opposite effect on improving memory retrieval in our animal model.

In contrast to in utero inflammatory stimulation, most studies addressing the effect of systemic maternal inflammation during pregnancy were shown to disrupt, not improve, the behavioral performance of exposed offspring in spatial learning and memory tasks [73,74,75,76,77]. However, an increase in memory retrieval in male offspring of dams injected intraperitoneally with staphylococcal endotoxin A was also documented [78]. This suggests that different mechanisms are involved in the regulation of these behavioral outputs dependent on how infectious agents or their ligands are localized and/or activate biological pathways within the placental–fetal compartment.

Disruptions in hippocampal neurogenesis can also lead to changes in other behavioral outputs, which were explored in this study, such as stress-induced anxiety response [65]. In particular, genetically induced alterations in neuronal production in the hippocampal dentate gyrus were shown to have an inverse effect on anxiety levels [31,79]. Therefore, the reduction in hippocampal neurogenesis in LPS-exposed pups [37] is consistent with our finding of increased anxiety in the OF test after exposure to intrauterine inflammation. In addition, our gene expression data show that prenatal inflammation can cause sex-specific changes in expression levels of various regulators of murine neurogenesis. These data shed some light on possible molecular mechanisms associated with hippocampal neurogenesis, which may underlie differential behavioral responses to prenatal immune stimulation in different sexes, such as heightened anxiety observed in mature male but not female pups following in utero inflammation. Our results show that inflammation-induced altered hippocampal gene expression occurs in several molecular pathways, including signaling cascades, such as Notch, BMP, Wnt/β-catenin, MAPK and Nrg1, transcriptional and/or chromatin regulation, neurotransmission, apoptosis, and App-associated pathology, among others. While most of these pathways are altered in both sexes, some of their molecular components showed changes specific to male or female hippocampi. For example, among several members of the Notch signaling pathway whose levels were elevated in response to in utero LPS treatment, *Delta-like 1* (*Dll1*) ligand and *Notch2* receptor were increased in females. The Notch signaling pathway is known to be involved in the regulation of multiple events during adult hippocampal neurogenesis [80]. Therefore, these differences in the downstream responses to inflammation-induced Notch pathway activation suggest differential effects on novel neuronal production in the hippocampi of male and female pups, which may, in turn, lead to alternative behavioral outcomes, such as those observed in this study. Our studies were limited by looking at a targeted panel of genes, but future more comprehensive gene expression studies are likely to reveal other pathways that are dysregulated by LPS throughout the lifespan.

In conclusion, we present some sex dichotomies with age after intrauterine inflammation in anxiety, memory, cognitive flexibility, and gene expression in the hippocampus. This work suggests that the interaction of age and sex is essential when considering behavior studies of intrauterine insults. Future studies will focus on understanding which pathways may be regulating the altered behavior and whether the gene expression differences persist into adulthood and continue to account for the observed behavioral differences. It is possible that these pathways may be targets for therapeutic interventions to improve long-term outcomes.

## 4. Materials and Methods

### 4.1. Mouse Model of Intrauterine Inflammation

An established mouse model of intrauterine inflammation [16] was used to study the effect of prenatal immune activation on specific behavioral outcomes in exposed offspring. Briefly, a mini-laparotomy was performed on CD-1 timed pregnant mice on E15. A single injection of *E. coli* LPS (055:B5, Sigma Aldrich, St. Louis, MO, USA; 50 μg/100 μL per animal) or saline (100 μL per animal) was applied to the right intrauterine horn between the first and second gestational sacs. For open field (OF) and Barnes maze (BM) experiments the pups delivered at term were culled to three pups per sex on P2-P3 and weaned on P21. Animals were maintained in a 12:12 h light:dark cycle and were allowed to feed ad libitum.

All animal care and treatment procedures were approved by the Institutional Animal Care Committee following the guidelines from the National Institutes of Health.

### 4.2. Behavioral Testing

Animal behavior was assessed in pups born at term using the following behavioral tests: ultrasonic vocalization (USV), open field (OF), and Barnes maze (BM). For USV testing, 11 male and female pups from different litters were tested per group (LPS or saline) on P5. USV testing was performed on separate cohorts from OF and BM. The OF test was performed using juvenile (P28) and mature (P67) pups of both sexes from LPS- and saline-exposed groups. One male and one female pup were tested per group per litter. Each experimental cohort was composed of three to five separate litters of each exposure for a total of LPS-treated males: *n* = 13; LPS-treated females: *n* = 13; saline-treated males: *n* = 12; saline-treated females: *n* = 12. The same juvenile and mature pups tested in OF were subsequently subjected to BM testing on P32–36 and P70–74, respectively. Animals were habituated to the testing area for at least 30 min prior to experimentation for OF and BM.

The USV test was performed on the offspring of saline- and LPS-injected dams at P5 according to a previously published protocol [81]. Briefly, USVs were recorded using an UltraSoundGate Condenser Microphone CM 16 (Avisoft Bioacoustics, Berlin, Germany) which was placed 12 cm above the testing surface within a sound attenuating cabinet. The recordings were made with Avisoft Recorder software (version 4.2.14; Avisoft Bioacoustics, Glienicke, Germany) using a sampling rate of 375,000 Hz in a 16-bit format. For signal analysis, fast Fourier transformation was performed with Avisoft SASLab Pro (version 4.40; Avisoft Bioacoustics, Glienicke, Germany) to generate spectrograms. Spectrograms were visually scanned to remove artifacts and the number of ultrasonic vocalizations (calls), average call duration, and the frequency of emitted sound were determined during a 5 min recording session.

To determine the effect of prenatal inflammation on exploration and anxiety responses, the OF testing was performed on pre-adolescent and mature pups of both sexes. At the beginning of each 10 min trial, mice were placed individually at the center of a round-shaped arena (San Diego Instruments, San Diego, CA, USA). A scaffold of infrared emitters and photodetectors was placed around the arena to detect beam breaks as the mouse moved. X*Y*-axis detectors collected peripheral and center beam breaks as their peripheral and central activities, respectively, and an elevated *Z*-axis detector collected vertical beam breaks as rearings. Rearings as well as central, peripheral, horizontal (the sum of central and peripheral activities), and total (the sum of rearings and horizontal activity) activities were recorded using the PAS–open field system (San Diego Instruments, San Diego, CA, USA). While most of these measurements were presented as the average number of beam breaks per a 10 min trial, central activity was expressed as a percentile from horizontal activity: (center activity/horizontal activity) × 100%.

To determine possible differences in spatial learning and memory between LPS- and saline-exposed groups, we conducted BM testing on male and female pups from both groups. BM was made out of a large, flat disc with twenty holes at the perimeter (San Diego Instruments, San Diego, CA, USA). All holes, except one escape hole, were blocked during acquisition trials. Distal landmark cues with unique geometric shapes were placed around the maze which was illuminated to ~1000 lux from an overhead halogen lamp. The escape hole was demarked by a specific visible cue. All trials were recorded using Microsoft Lifecams (Microsoft, Redmond, VA, USA) for documentation and offline analysis. The procedure consisted of four acquisition sessions performed on four consecutive days, specifically, P32–35 and P70–73 for pre-adolescent and mature animals, respectively, followed by a probe trial after acquisition. Two acquisition trials per day were performed with a 30 min interval. If the escape compartment was entered, the trial ended and the latency to escape was recorded. If a mouse did not enter the escape compartment in 150 s, it was gently guided into the hole.

Twenty-four hours after the last acquisition trial, on P36 and P74, respectively, a two-minute probe trial was performed with all holes occluded, including the escape compartment. Image analysis software (ANYmaze, Stoelting Co., Wood Dale, IL, USA) was used to divide the maze into four equal quadrants virtually, and the time spent in each quadrant was recorded. Mice that learned to associate the cued quadrant with the escape compartment location spent more time in the quadrant where the cue was located.

### 4.3. Hippocampal Dissections

Hippocampal dissections were performed on P7 brains isolated from male and female offspring of LPS- and saline-treated dams as published [82].

### 4.4. Gene Expression Analysis Using Mouse Neurogenesis Arrays

Prior to analyzing alterations in gene expression associated with mouse neurogenesis, the sex of each pup was determined by quantifying male- and female-specific transcripts, SRY and XIST, respectively, in RNA samples isolated from pups’ tails. Tissue homogenization was performed using TissueLyser II (QIAgen Sciences, Germantown, MD, USA) and a standard protocol for RNA extraction with QIAzol Lysis reagent (QIAgen Sciences, Germantown, MD, USA) and 1-bromo-3-chloropropane reagent (BCP, Thermo Fisher Scientific, Philadelphia, PA, USA) was applied. After assigning information about sex for each sample, all samples were subdivided into the following four groups: LPS-treated female samples (*n* = 13), LPS-treated male samples (*n* = 10), saline-treated female samples (*n* = 5), and saline-treated male samples (*n* = 5).

Subsequently, total RNA was isolated from the hippocampi of male and female pups exposed to prenatal LPS or saline using the RNeasy Microarray Tissue mini kit (QIAgen Sciences, Germantown, MD, USA). The integrity of RNA samples was confirmed using 2100 Bioanalyzer (Agilent Genomics, Santa Clara, CA, USA) and cDNAs were synthesized using the RT^2^ First Strand Kit (Qiagen Sciences, Germantown, MD, USA). Gene expression was compared between the hippocampal samples from LPS- and saline-exposed pups using Mouse Neurogenesis Arrays (RT^2^ Profiler PCR Array, Qiagen Sciences, Germantown, MD, USA). Expression levels of 84 genes with well-documented roles in murine neurogenesis were examined. qPCR amplification was done using RT^2^ SYBR Green ROX qPCR Mastermix (Qiagen Sciences, Germantown, MD). All RT-PCR reactions were repeated four times and changes in gene expression were analyzed using the ΔΔC_T_ method. 

Differentially expressed genes for each sex were entered into GeneMania to determine functional networks regulated by intrauterine inflammation in the hippocampus [83] (https://genemania.org, blue version, accessed 20 May 2021). The top five pathways were noted for each sex.

### 4.5. Statistical Analysis

Statistics were performed as described previously using R-studio [58]. Briefly, we used mixed models with generalized estimating equations in the geepack package in R with cohort as the random effect [84]. Linear mixed-effects models in the lme4 package in R was used for BM since repeated measures between trials were required [85]. Statistical significance is shown for LPS-exposure, sex, and interaction between LPS exposure and sex since intrauterine inflammation has been shown to have sex-dichotomous effects [21]. Statistical significance was set at * *p* < 0.05, ** *p* < 0.01, and *** *p* < 0.001. We corrected *p*-values from mixed model analyses with a Benjamini–Hochberg correction.

Gene expression data generated using Mouse Neurogenesis Arrays were analyzed on the RT^2^ Profiler PCR Array Data Analysis Webportal (QIAgen Sciences, Germantown, MD, USA) using the ΔΔC_T_ method. Target genes were normalized to three housekeeping genes: β-Glucuronidase (*Gusb*), β-2 microglobulin (*B2m*), and Heat shock protein 90 α (cytosolic), class B member 1 (*Hsp90ab1*). The replicate 2^(−ΔCT)^ values were compared for each target gene in LPS- and saline-treated groups using Student’s *t*-test (two-tail distribution with equal variances between the two groups of samples). We corrected *p*-values from mixed model analyses with a Benjamini–Hochberg correction. Male and female datasets were analyzed separately. The corrected *p*-values that are less than 0.10 were considered significant. The fold change in gene expression levels between LPS- and saline-treated groups were calculated as 2^(−ΔΔCT)^.

## Data Availability

All data generated or analyzed during this study are included in this article. Further inquiries can be directed to the corresponding author.
